# Transmission of COVID-19 and its Determinants among Close Contacts of COVID-19 Patients

**DOI:** 10.34172/jrhs.2021.48

**Published:** 2021-04-25

**Authors:** Reyhaneh Jashaninejad, Amin Doosti-Irani, Manoochehr Karami, Fariba Keramat, Mohammad Mirzaei

**Affiliations:** ^1^Department of Epidemiology, School of Public Health, Hamadan University of Medical Sciences, Hamadan, Iran; ^2^Research Center for Health Sciences, Hamadan University of Medical Sciences, Hamadan, Iran; ^3^Brucellosis Research Center, Hamadan University of Medical Sciences, Hamadan, Iran; ^4^Department of Infectious Disease, Sina Hospital, Hamadan University of Medical Sciences, Hamadan, Iran; ^5^Deputy for Public Health, Hamadan University of Medical Sciences, Hamadan, Iran

**Keywords:** SARS-CoV-2, COVID-19, Contact tracing, Secondary attack rate, Close contacts

## Abstract

**Background:** This study aimed to determine the secondary attack rate (SAR) and its determinants to describe the clinical features and epidemiological aspects of patients and determine the risk factors of COVID-19 among household contacts in Hamadan Province, west of Iran.

**Study design:** A cohort study.

**Methods:** In this cohort study, a total of 323 index cases and 989 related close contacts ages more than 15 years old (family members, relatives, and co-workers) were enrolled using a manual contact tracing approach, and all participants were tested by reverse transcription polymerase chain reaction test. In this research, the frequency of symptoms was assessed, the SAR among contacts of index cases was calculated, and the risk factors of COVID-19 were evaluated by the logistic regression model.

**Results:** The secondary attack rate for total household members of index cases was estimated at 31.7% (95% CI: 28.8-34.7). It was found that among household contacts, the highest SARs were related to spouses 47.1% (95% CI: 38.7-55.7) and grandparents/parents 39.3% (95% CI: 29.4, 49.9) of index cases, who had also higher risks to become secondary cases (adjusted odds ratio [OR]=2.98, 95% CI: 1.31-6.75 and adjusted OR=2.76, 95% CI: 1.18-6.44, respectively). Considering the occupation of contacts, unemployed and retired people and housewives were most susceptible for transmission of COVID-19. It was revealed that cough was the most prevalent symptom among index and secondary cases.

**Conclusions:** Our findings indicated that spouses and grandparents/parents of index cases were the most susceptible individuals for COVID-19 transmission. Prolonged exposure with index case before COVID-19 diagnosis raised the chance of infection among secondary cases.

## Introduction

By late December 2019, a new member of the coronaviruses family named Coronavirus disease -2019 (COVID-19) emerged in Wuhan, China, and spread rapidly around the world^1^. The most common symptoms include fever, difficulty breathing or dyspnea, fatigue, and cough that can contribute to pneumonia ^2,3^. After months since the diagnosis of the first cases of COVID-19, the confirmed cases were estimated at more than 49,000,000 cases and over 1,240,000 deaths globally and 673,250 affected cases and 37,832 deaths in Iran until 8 November 2020. The number of patients and deaths is increasing every day encountering global health with a serious problem ^4^.

All people in different age groups may be infected by COVID-19; however, the vulnerability and mortality of older people with comorbidity and chronic diseases, such as heart disease, diabetes, and hypertension, are higher during this pandemic ^5^. The results of studies indicate that COVID-19 is a contagious and symptom-free disease and can be transmitted person to person by patients’ droplets without any symptoms in many cases. Regarding this, these patients can be regarded as one of the potential sources of spreading the infection ^6,7^. Household and close contacts in family and among relatives and family clustering are some of the main causes of novel coronavirus spread. Therefore, focusing on family clustering and close contacts can be one of the effective factors to control the disease^8,9^. Based on the findings of other studies, the secondary attack rate (SAR) is more common among adults and symptomatic cases are more responsible for secondary cases than asymptomatic cases ^10,11^.

 The first cases of COVID-19 were reported in Iran by mid-February, 2020. This study aimed to determine the SAR and its determinants, describe the clinical features and epidemiological aspects of patients, and determine the risk factors of COVID-19 among close and household contacts in Hamadan Province, Iran.

## Methods

In this cohort study, all data were collected from rural/urban comprehensive primary health centers (PHC centers) in Hamadan Province, west of Iran, from mid-May to mid-July, 2020. This research used a manual contact tracing approach and investigated suspected people referred to these centers and confirmed them by reverse transcription polymerase chain reaction (RT-PCR) test. The data of positive laboratory-confirmed cases of COVID-19 and their close contacts were collected and traced. Following contact tracing of related close contacts, they were invited to be tested at PHC centers. Close contact is defined as a person who had exposure or lived with a probable or confirmed case or had direct and face-to-face contact 2 days before and 14 days after exposure with the index case. Suspected COVID-19 cases are defined as cases who showed clinical symptoms, such as fever, cough, fatigue, and another related symptom, or had close contact with a positive COVID-19 case ^1^. The index case is determined as the first case of COVID-19 in the family confirmed by laboratory test ^13^. People older than 15 years old were included in this study. The inclusion criteria were cases diagnosed with a positive RT-PCR test and asymptomatic individuals with a positive test.

 The instruments used in this research to collect the required data were two questionnaires. The first questionnaire used for index case included demographic characteristics, such as age, gender, occupation, weight, height, and the number of people in contact with the infected patients, and clinical features, such as symptoms and signs, underlying disease, hospitalization, and outcome (recovery, death by COVID-19, and death by other diseases).

 The second questionnaire, for their contacts, consisted of three parts, including demographic characteristics (age, gender, occupation, weight, height, relationship with patient), clinical features (symptoms and signs, underlying disease, history of close contacts, time of close contact before and after affection to COVID-19 in a day), and the result of RT-PCR test for all contacts. Eligible close contact was defined as a family or a cluster with one member as an index patient. All patients and their close contacts were confirmed by the RT-PCR test. A total of 323 index cases with 989 family contacts (members of nuclear and immediate family, colleagues, and neighbors) were enrolled in the study. Some variables, such as height and weight, were self-reported, while clinical symptoms were checked by a physician.

###  Statistical analysis

The SAR is defined as a proportion of infected cases from the total of household contacts that had close contact with index cases^14,15^. The SAR was calculated using the following formula ^16^:


$$SAR=\frac{\mathrm{Number}\;\mathrm{of}\;\mathrm{secondary}\;\mathrm{infected}\;\mathrm{contacts}}{\mathrm{Total}\;\mathrm{number}\;\mathrm{of}\;\mathrm{contacts}}\times 100$$

The association of possible risk factors and positive RT-PCR test was assessed by the logistic regression model. Variables not significant in a univariable model were omitted in the multivariable model. The data were analyzed in STATA software (version 16), and the results were reported at a 95% confidence interval (CI).

## Results

The statistical population of this study consisted of 1,312 participants, including 323 index cases and 989 close contacts, among which 314 contacts had positive RT-PCR test. The SAR of all 989 household contacts was estimated at 31.7% (95% CI: 28.8-34.7). The most SARs among household contacts with index case were obtained at 47.1% (95% CI: 38.7-55.7) to spouses and 39.3% (95% CI: 29.4-49.9) to parents and grandparents. The SARs among close contacts related to their job were calculated at 58.8% (95% CI: 32.9-81.5), 35.2% (95% CI: 22.4-49.9), 34.4% (95% CI 29.7, 39.3) for unemployed people, retired people, and housewives, respectively. Regarding the duration of close contacts before the diagnosis of the disease in index cases within 2 weeks, it was found that SARs for those close contacts with more than one-hour daily contacts and one-hour or less daily contacts with index case were 35.8% (95% CI: 32.5, 39.3) and 15.9% (95% CI: 11.0, 21.9), respectively (Table 1).

**Table 1:** Percentage of secondary attack rate among close contacts of index cases

**Variables**	**Total contacts**	**Infected**	**Secondary attack rate % (95% CI)**
General secondary transmission	989	314	31.7 (28.8-34.7)
Gender			
Male	479	146	30.4 (26.3-34.8)
Female	510	168	32.9 (28.8-37.2)
Age (year)			
15-24	110	35	31.8 (23.2-41.3)
25-34	224	68	30.3 (24.4-36.8)
35-44	250	81	32.4 (26.6-38.5)
45-54	191	59	30.8 (24.4-37.9)
55-64	110	38	34.5 (25.7-44.2)
65-74	52	17	32.6 (20.3-47.1)
≥75	29	11	37.9 (20.6-57.7)
Missing	23	5	
Body mass index (kg/m^2^)			
<24.99	475	135	28.4 (24.4-32.7)
25-29.99	362	125	34.5 (29.6-39.6)
≥30	127	48	37.7 (29.3-46.8)
Missing	25	6	
Relationship with the index patient
Grandchild	67	11	16.4 (8.4-27.4)
Sister/brother	61	15	24.5 (14.4-37.2)
Child	299	100	33.4 (28.1-39.1)
Grandmother, grandfather mother, father	94	37	39.3 (29.4-49.9)
Spouse	142	67	47.1 (38.7-55.7)
Daughter-in-law	98	26	26.5 (18.1-36.4)
Others (aunt, colleague uncle, neighbor)	228	58	25.4 (19.9-31.6)
Job			
Workman	56	9	16.0 (7.6-28.3)
Employed	136	37	27.2 (19.9-35.4)
Housewife	395	136	34.4 (29.7-39.3)
Retired	51	18	35.2 (22.4-49.9)
Self-employed	225	73	32.4 (26.3-38.9)
Student	45	12	26.6 (14.6-41.9)
Unemployed	17	10	58.8 (32.9-81.5)
Others	58	18	31.0 (19.5-44.5)
Missing	6	1	
Contact with index case 2 weeks before diagnosis
≤1 hour (daily)	188	30	15.9 (11.0-21.9)
>1 hour (daily)	780	280	35.8 (32.5-39.3)
Missing	21	4	
Contact with index case during treatment
No	417	105	25.1 (21.0-29.6)
Yes	546	201	36.8 (32.7-41.0)
Missing	26	8	
Contact with the index case after discharge and during convalescence
≤1 hour (daily)	810	243	30.0 (26.8-33.2)
>1 hour (daily)	150	65	43.3 (35.2-51.6)
Missing	29	6	
Use sharing equipment during convalescence
No	692	215	31.0 (27.6-34.6)
Yes	260	82	31.5 (25.9-37.5)
Missing	37	17	

In this study, out of 323 index cases, 72, 222, and 26 cases underwent outpatient treatment, hospitalized in the ward, and hospitalized in the intensive care unit (ICU), respectively. More than 50% of total index cases and hospitalized cases in the ward and ICU were obese and overweight. Moreover, 42.7% of total index cases and approximately 70% of index cases hospitalized in the ICU had at least one underlying disease (Table 2). In addition, 87.6% (283) of index cases had one or more clinical symptoms, while 12.4% (40) of them were symptom-free. The most frequent symptoms among index cases were reported to be cough (65.0%), difficulty breathing (60.7%), and sore throat (27.9%). Regarding the contacts with positive RT-PCR test 68.5% (n=215), the most common symptoms were found to be cough (30.3%), joint and muscle soreness (17.5%), headache (16.9%), fever (14.3%), and fatigue (14.0%) (Table 3).

**Table 2:** Baseline characteristics of index cases and close contacts

**Variables **	**Contacts with positive cases ** **(RT-PCR; n=314)**		**Index cases (n=323)**		**Index cases by group, n (%)**					
					**Outpatients (n=72)**		**Hospitalized in ** **the ward (n=222)**		**Hospitalized in the** **ICU (n=26)**	
	**Number**	**Percent**	**Number**	**Percent**	**Number**	**Percent**	**Number**	**Percent**	**Number**	**Percent**
Sex										
Female	168	53.5	170	52.6	34	47.2	126	56.8	8	30.8
Male	146	46.5	153	47.4	38	52.8	96	43.2	18	69.2
Age (year)										
15-24	35	11.1	5	1.5	2	2.8	3	1.4	0	0.0
25-34	68	21.7	46	14.2	23	31.9	19	8.6	4	15.4
35-44	81	25.8	46	14.2	14	19.4	30	13.5	0	0.0
45-54	59	18.8	41	12.7	9	12.5	31	14.0	1	3.8
55-64	38	12.1	57	17.6	7	9.7	44	19.8	5	19.2
65-74	17	5.4	56	17.3	8	11.1	42	18.9	6	23.1
≥75	11	3.5	62	19.2	7	9.7	47	21.2	8	30.8
Missing	5	1.6	10	3.1	2	2.8	6	2.7	2	7.7
Body mass index (kg/m^2^)									
< 24.9	135	43.0	144	44.6	37	51.4	94	42.3	11	42.3
25-29.9	125	39.9	128	39.6	27	37.5	91	41.0	9	34.6
≥30	48	15.3	46	14.2	8	11.1	32	14.4	6	23.1
Missing	6	1.9	5	1.6	0	0.0	5	2.2	0	0.0
Underlying disease									
Yes	61	19.4	138	42.7	25	34.7	94	42.3	18	69.3
No	252	80.3	163	50.5	40	55.6	116	52.3	5	19.2
Missing	1	0.3	22	6.8	7	9.7	12	5.4	3	11.5
Clinical symptoms									
Yes	215	68.5	283	87.6	60	83.3	196	88.3	24	92.3
No	99	31.5	40	12.4	12	16.7	26	11.7	2	7.7

RT-PCR: Reverse transcription polymerase chain reaction

**Figure 1:**
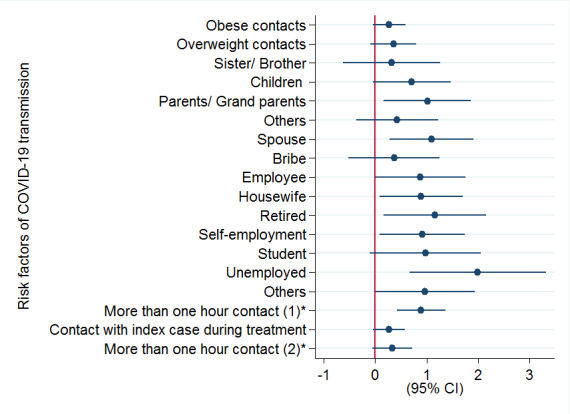
Determinants of COVID-19 transmission among secondary cases

It was revealed that among close contacts of index cases, the highest risk rates of infection to become secondary cases were related to spouses (adjusted odds ratio [OR]=2.98, 95% CI: 1.31-6.75) and parents/grandparents of index cases (adjusted OR=2.76, 95% CI: 1.18, 6.44), compared to grandchildren (as a group with the lowest rate). Based on job categorical contacts of index cases, unemployed people (adjusted OR=7.37, 95% CI: 1.95, 27.75), retired people (adjusted OR=3.18, 95% CI: 1.17, 8.65), and housewives (adjusted OR= 2.44, 95% CI: 1.08, 5.50) had the most risk of infection than workmen (reverence group). According to the findings, contacts who had more than one-hour daily contact with the index case, before the diagnosis of the disease in index cases had a higher risk of infection (adjusted OR=2.44, 95% CI: 1.52, 3.93), compared to contacts who had one-hour and less close contact (Table 4, Figure 1).

The results of this study indicated that the most commonly observed symptoms among index cases were cough, dyspnea, and sore throat (65.0%, 60.7%, and 27.9% respectively). It was revealed that among close contacts with positive RT-PCR test, the symptoms of cough, joint and muscle soreness, headache, fever, and fatigue were more frequent. These results are in line with those of similar studies performed in Iran and China ^20-22^. The findings of another systematic review and meta-analysis study confirmed fever (78%), cough (57%), and fatigue (31%) as the most common symptoms from 9 countries^23^. The results of this study and other studies are approximately similar; however, in our study sore throat and muscle soreness were common and fever was less prevalent, in comparison to the symptoms reported in other studies. It seems the reasons for these differences are related to various definitions, genetic differences, or different immune system responses in varied places.

Based on the results of the present research, the SARs were higher in contacts aged 55-64 and over 75 years than in other age groups, which was approximately in line with those reported in a study from Taiwan^24^. Among household contacts, the spouses and parents/grandparents of index cases had the highest SAR and risk of infection. The findings of available studies from Zhejiang and Wuhan, China, indicated that the highest SAR among household contacts was related to spouses of index cases. Considering these results found in their and our studies, spouses of index cases might be more vulnerable to become infected because of their more daily close contact ^19,25^. The researchers of the current study also collected information about participants' jobs and found that the SAR was significantly higher in unemployed and retired people and housewives than in others. The reason for this might be due to the fact that unemployed and retired people stay longer at home and have more exposure and close contact with index cases and family members.

(1) *^*^= Daily hours of contact with index cases 2 weeks before the disease diagnosis in index cases

(2)*^*^= Daily hours of contact with index cases after discharge and during convalescence

**Table 3:** Clinical characteristics of index cases and close contacts with positive RT-PCR test

**Variables**	**Close contacts with** **positive (RT-PCR)** **(n=314) **		**Index cases** **(n=323) **	
	**Number **	**Percent**	**Number**	**Percent**
Clinical symptoms				
Yes	215	68.5	283	87.6
No	99	31.5	40	12.4
Chillness				
Yes	36	11.5	3	0.9
No	278	88.5	320	99.1
Vomiting				
Yes	17	5.4	2	0.6
No	297	94.6	321	99.4
Nasal congestion				
Yes	13	4.1	10	3.1
No	301	95.1	313	96.9
Difficulty breathing (dyspnea)			
Yes	33	10.5	196	60.7
No	281	89.5	127	39.3
Diarrhea				
Yes	15	4.8	19	5.9
No	299	95.9	304	94.1
Runny nose				
Yes	18	5.7	25	7.7
No	296	94.3	298	92.3
Joint and muscle soreness				
Yes	55	17.5	1	0.3
No	259	82.5	322	99.7
Loss of taste and smell				
Yes	25	8.0	4	1.2
No	289	92.0	320	98.8
Headache				
Yes	53	16.9	2	0.6
No	261	83.1	322	99.4
Fever				
Yes	45	14.3	7	2.2
No	269	85.7	316	97.8
Fatigue				
Yes	44	14.0	2	0.6
No	270	86.0	321	99.4
Cough				
Yes	95	30.3	210	65.0
No	219	69.7	113	35.0
Sore throat				
Yes	51	16.2	90	27.9
No	263	83.8	233	72.1

RT-PCR: Reverse transcription polymerase chain reaction

## Discussion

 Findings show a higher proportion of SAR among household contacts in comparison to other close contacts. It was also found that spouses of index cases were more vulnerable than other household contacts for secondary infection. The researchers of this study estimated that the secondary cases with higher hours of contact before the diagnosis of the disease in index cases had a higher SAR.

In our study, the SAR in total close household contacts was obtained at 31.7% (95% CI: 28.8-34.7), which was considerably lower and a little more than the SARs reported in studies conducted in Guangzhou and Wuhan, China (49.56% and 30%, respectively)^17,18^, and about similar to family SAR reported from Zhejiang Province, China, (31.6%) ^19^. This rate was substantially higher than the SAR reported from Castellon, Spain, (%11.1) ^14^. These discrepancies in reported SAR can be attributed to different health behaviors, health protocols, use of different personal protective equipment, and the time of the diagnosis of the disease in index cases. Another considerable point can be the culture of people of the countries. In Iran, family members and relatives have strong family ties and are constantly in close contact with each other. Additionally, economic problems in Iran did not allow further restrictions or led to the failure of the imposed restrictions.

Our finding showed a significant association between increasing hours of contacts and infection to disease 2 weeks before the diagnosis of the disease in the index case, indicating that the infection might be transmitting before diagnosis and symptomatic period ^26,27^. Therefore, based on these results, longer contact and exposure can increase the risk of transmission of infection. In this research, contact with index cases during treatment showed no significant association with after discharge and during convalescence, which might be because of using masks or implementing protective measures. This study had several limitations, and no information was available about the quantity and quality of using masks and implementing protective measures. However, the researchers knew that comprehensive health centers provided healthy packs for index cases and their positive contacts. Since the researchers did not have any information about the home space of index cases, there was a possibility of selection bias in our study because it was possible that some of the close contacts had not been referred for the RT-PCR test. In this study, subgroup analysis was not performed between local and imported cases, and information about imported cases was defective and inaccessible.

**Table 4:** Risk factors of COVID-19 transmission among secondary cases

**Variables**	**Total contacts**	**Infected**	**Crude** **OR (95% CI)**	**P-value**	**Adjusted** **OR (95% CI)**	**P-value**
Gender						
Male	479	146	1.00		-	-
Female	510	168	1.12 (0.85-1.46)	0.406	-	-
Age (year)						
15-24	110	35	1.07 (0.65-1.75)	0.786	-	-
25-34	224	68	1.00			-
35-44	250	81	1.09 (0.74-1.62)	0.632	-	-
45-54	191	59	1.02 (0.67-1.55)	0.907	-	-
55-64	110	38	1.21 (0.74-1.96)	0.440	-	-
65-74	52	17	1.11 (0.58-2.12)	0.743	-	-
≥75	29	11	1.40 (0.62-312)	0.409	-	-
Missing	23	5			-	-
Body mass index (kg/m^2^)						
<24.99	475	135	1.00		1.00	
25-29.99	362	125	1.32 (0.98-1.78)	0.059	1.31 (0.95-1.80)	0.091
≥30	127	48	1.53 (1.01-2.30)	0.042	1.42 (0.91-2.21)	0.120
Missing	25	6				
Relationship with the index patient						
Grandchild	67	11	1.00		1.00	
Sister/brother	61	15	1.66 (0.69-3.96)	0.254	1.37 (0.53-3.55)	0.514
Child	299	100	2.55 (1.28-5.09)	0.008	2.03 (0.95-4.34)	0.065
Grandmother, grandfather, mother, father	94	37	3.30 (1.53-7.11)	0.002	2.76 (1.18-6.44)	0.019
Spouse	142	67	4.54 (2.20-9.39)	0.001	2.98 (1.31-6.75)	0.009
Daughter-in-law	98	26	1.83 (0.83-4.03)	0.129	1.44 (0.59-3.50)	0.416
Others	228	58	1.73 (0.85-3.53)	0.128	1.53 (0.68-3.40)	0.297
Job						
Workman	56	9	1.00		1.00	
Employee	136	37	1.95 (0.87-4.37)	0.104	2.41 (1.00-5.84)	0.050
Housewife	395	136	2.74 (1.30-5.76)	0.008	2.44 (1.08-5.05)	0.031
Retired	51	18	2.84 (1.14-7.11)	0.025	3.18 (1.17-8.65)	0.023
Self-employed	225	73	2.50 (1.16-5.39)	0.019	2.49 (1.09-5.71)	0.030
Student	45	12	1.89 (0.71-5.02)	0.196	2.65 (0.90-7.82)	0.077
Unemployed	17	10	7.46 (2.24-24.78)	0.001	7.37 (1.95-27.75)	0.003
Others	58	18	2.35 (0.95-5.80)	0.064	2.64 (1.00-6.98)	0.049
Missing	6	1				
Contact with index case 2 weeks before diagnosis					
≤1 hour (daily)	188	30	1		1	
>1 hour (daily)	780	280	2.94 (1.94-4.47)	0.001	2.44 (1.52-3.93)	0.001
Missing	21	4				
Contact with index case during treatment						
No	417	105	1		1	
Yes	546	201	1.73 (1.30-2.29)	0.001	1.31 (0.95-1.79)	0.089
Missing	26	8				
Contact with index case after discharge and during convalescence				
≤1 hour (daily)	810	243	1		1	
>1 hour (daily)	150	65	1.78 (1.24-2.54)	0.001	1.39 (0.94-2.06)	0.093
Missing	29	6				
Use sharing equipment during convalescence					
No	692	215	1		-	
Yes	260	82	1.02 (0.75-1.38)	0.889	-	-
Missing	37	17				

## Conclusions

 According to the results, spouses and grandparents/parents of index cases were the most susceptible people for the transmission of COVID-19. Moreover, prolonged exposure with index case before COVID-19 diagnosis raised the chance of spreading infection among secondary cases.

## Acknowledgment

 This study was derived from an MSc thesis submitted to the Hamadan University of Medical Sciences, Hamedan Province, Iran.

## Conflict of interest

 The authors declare that there is no conflict of interest.

## Funding

 The study was partially funded by the Vice-Chancellor for Research and Technology, Hamadan University of Medical Sciences (Grant No. 9905213283). The funder had no role in study design, data collection and analysis, decision to publish, or preparation of the manuscript.

## Highlights

- The secondary attack rate (SAR) among total household contacts was 31.7% (95% CI: 28.8-34.7).
- Among household members, spouses and parents/grandparents of index cases had the highest SAR and risk to become secondary cases.
- Among close contacts, based on their job, unemployed and retired people and housewives had higher SAR and were also more susceptible for the transmission of COVID-19.
- Contact with index case 2 weeks before the disease diagnosis of index case increased the risk of infection among secondary cases.
- Cough was the most frequent symptom among both index cases and their close contacts.
